# Self-Consistent Study of the Electronic Properties of a Single Exciton in Nanoscopic CdSe Quantum Dots

**DOI:** 10.3390/nano16191266

**Published:** 2026-10-07

**Authors:** J. A. Gil-Corrales, C. A. Dagua-Conda, J. A. Vinasco, J. Sierra-Ortega, Gene Elizabeth Escorcia-Salas, A. L. Morales, D. B. Hayrapetyan, C. A. Duque

**Affiliations:** 1 Facultad de Ciencias, Instituto Tecnológico Metropolitano (ITM)-Institución Universitaria, Campus Fraternidad, Calle 73 No. 76A-354 Vía al Volador, Medellín 050034, Colombia; 2 Grupo de Materia Condensada-UdeA, Instituto de Física, Facultad de Ciencias Exactas y Naturales, Universidad de Antioquia UdeA, Cl 70 No. 52-21, Medellín 050010, Colombia; carlos.dagua@udea.edu.co (C.A.D.-C.); alvaro.morales@udea.edu.co (A.L.M.); 3 Grupo de Investigación Ce-Lab, Departamento de Ciencias Básicas, Facultad de Ingeniería y Administración, Universidad Nacional de Colombia, Palmira 763533, Colombia; javinascos@unal.edu.co; 4 Grupo de Investigación en Teoría de la Materia Condensada, Departamento de Física, Universidad del Magdalena, Santa Marta 470004, Colombia; jcsierra@unimagdalena.edu.co (J.S.-O.); gescorcia@unimagdalena.edu.co (G.E.E.-S.); 5 Quantum Materials and Nanophotonics Laboratory, A.B. Nalbandyan Institute of Chemical Physics NAS RA, P. Sevak 5/2, Yerevan 0014, Armenia; david.hayrapetyan@rau.am; 6 Russian-Armenian University, 123 Hovsep Emin Str., Yerevan 0051, Armenia

**Keywords:** quantum dots, excitons, coulomb interaction, self-consistent

## Abstract

The electronic and excitonic properties of a single exciton confined in nanoscopic CdSe quantum dots with different geometries are investigated using a self-consistent Schrödinger-Poisson approach within the effective mass and Hartree approximations. Box, cylindrical, ellipsoidal, and square-based pyramidal quantum dots are considered, keeping their volume and cross-sectional area equal to isolate geometrical effects. The results show that increasing either the longitudinal ($L_{z}$) or transverse (*L*) dimension reduces the carrier confinement energies and produces a redshift of the exciton transition, with a stronger effect when the transverse dimensions are increased. The electron-hole Coulomb interaction decreases as the carrier wave functions become more spatially extended and remains strongly dependent on the quantum-dot geometry. Notably, the pyramidal structure exhibits the most pronounced geometry-dependent behavior, while the cylinder and ellipsoid show similar exciton transition energies because the energy associated with the strong confinement in the xy plane dominates over that associated with the weaker confinement along the *z* direction as the longitudinal parameter is increased. Similarly, the box and cylinder exhibit comparable exciton transition energies as the transverse parameter is increased. These results demonstrate that the shape and aspect ratio of nanoscopic quantum dots provide effective parameters for controlling their excitonic and optical properties.

## 1. Introduction

Advanced experimental techniques such as molecular beam epitaxy (MBE) and metal-organic chemical vapor deposition (MOCVD) have enabled fine control over the synthesis and growth of semiconductor nanostructures [1,2,3,4,5,6,7,8,9]. These systems have become central to materials physics and nanotechnology because their electronic properties can be controlled by reducing their dimensions [10,11,12]. This effect is most pronounced in the strong quantum confinement regime, which is reached when the nanocrystal radius is smaller than the exciton Bohr radius. In this regime, quantum effects govern charge-carrier behavior, and the bulk continuum of states transforms into discrete energy levels. This discretization, tunable through the size and shape of the nanostructure, forms the basis for designing its electronic and optical properties [13,14,15,16,17,18]. In this context, the II–VI semiconductor CdSe, whose emission can be tuned across the entire visible spectrum by controlling its size [19,20]. This sensitivity of the electronic and optical properties to size has established CdSe nanostructures as a platform for potential applications in modern optoelectronics, including light-emitting diodes (LEDs) with high color purity [21], ultrasensitive photodetectors [22], next-generation solar cells [23], and single-photon emitters [24], key to quantum information technologies.

The optical response of CdSe nanostructures is fundamentally determined by the formation and dynamics of the exciton [25,26,27], a quasiparticle consisting of an electron-hole pair (e−h) bound by the Coulomb interaction. In the strong quantum confinement regime, reducing the size of the nanostructure significantly alters the exciton binding energy, modulating the transition energies that govern the absorption and emission spectra [28,29,30,31,32,33]. The pioneering work of Efros and Efros [34] proposed the first model of exciton quantum confinement in microcrystals, establishing the framework for classifying confinement regimes according to the relationship between the radius of a semiconductor sphere (*R*) and the characteristic Bohr radii ($a_{B}$). The authors showed that when $R\ll a_{B}$, the confinement energy dominates over the Coulomb interaction, such that the latter can be treated as a perturbation within the effective-mass approximation. The effective mass approximation is a robust and computationally efficient framework that accurately reproduces the electronic structure of the nanostructure [35].

Although the general effective-mass approximation performs well, describing the electron–hole Coulomb interaction requires a more rigorous treatment for modeling these systems. The Coulomb potential is commonly addressed with varying levels of rigor, ranging from complete neglect to perturbative treatment and even self-consistent schemes. For example, Bludov and Vasilevskiy [36], when modeling excitons confined in spherical CdSe quantum dots (QDs), adopt the strong confinement limit and neglect the Coulomb interaction between the electron and the hole, so that the excitonic spectrum is determined solely by the confinement kinetic energy. In a more refined treatment, Pellegrini et al. [37] present a comparative theoretical and experimental study of type-II heterostructures with a finite-depth square potential well. They obtain the electron and hole envelope functions analytically, neglecting the Coulomb interaction, and subsequently incorporate the electrostatic coupling term through first-order perturbation theory. However, Şahin et al. [38] implemented a self-consistent Hartree scheme and showed that the Coulomb interaction modifies the charge density, alters the wave-function distribution, and induces band bending. Since the oscillator strength and optical properties depend directly on the spatial overlap between the electron and hole envelopes, a formalism that accounts for these modifications of the wave functions is required.

Numerous theoretical studies on confined excitons assume spherical symmetry, including the self-consistent scheme discussed above [39,40,41,42]. However, real semiconductor nanostructures, obtained using modern epitaxial and colloidal synthesis techniques [43], adopt diverse shapes such as pyramids obtained through Stranski–Krastanov growth [44], nanowires [45], nanoplatelets [46], and nanotubes [47]. The shape and aspect ratio modify the confinement potential profile, altering the energy spectrum and optical response of the nanostructure. In previous work [48], we showed how geometry determines the thermodynamic properties of non-interacting particles in QDs of four shapes (box, cylinder, ellipsoid, and pyramid), while keeping the volume and cross-sectional area constant to isolate the effect of shape. However, to the best of our knowledge, no previous work has examined, within a self-consistent scheme, how geometry modulates exciton physics when the total volume is held fixed. In the present study, we fill this gap by solving the self-consistent Schrödinger–Poisson problem for four distinct geometries, box, cylinder, ellipsoid, and square-based pyramid, compared at equal volume and equal cross-sectional area, so that the differences observed can be attributed to shape alone rather than to size.

In this work, we study the electronic and excitonic properties of a single exciton confined in nanoscopic CdSe QDs with four geometries: box, cylinder, ellipsoid, and square-based pyramid. Within the effective mass and Hartree approximations, we self-consistently solve the Schrödinger and Poisson equations using the finite element method (FEM). By assuming the same volume and cross-sectional area in all structures, we can isolate the effect of shape and attribute the observed differences solely to geometry rather than size. By varying the transverse (*L*) and longitudinal ($L_{z}$) dimensions, the system evolves from zero-dimensional (0D) confinement to a quasi-one-dimensional (1D) quantum wire and, finally, to a quasi-two-dimensional (2D) nanoplatelet. The theoretical procedure is compared with experimental absorption data reported by Ekimov et al. [49] for spherical CdSe QDs. The results show that the Coulomb interaction depends on the shape even at constant volume and cross-sectional area and that the excitonic transition energy is determined by the competition between confinement and electron-hole attraction. These results indicate that the spatial distribution of volume is as important as the volume itself in determining the nanostructure’s excitonic properties.

## 2. Theory

### 2.1. The Model

**Figure 1:**
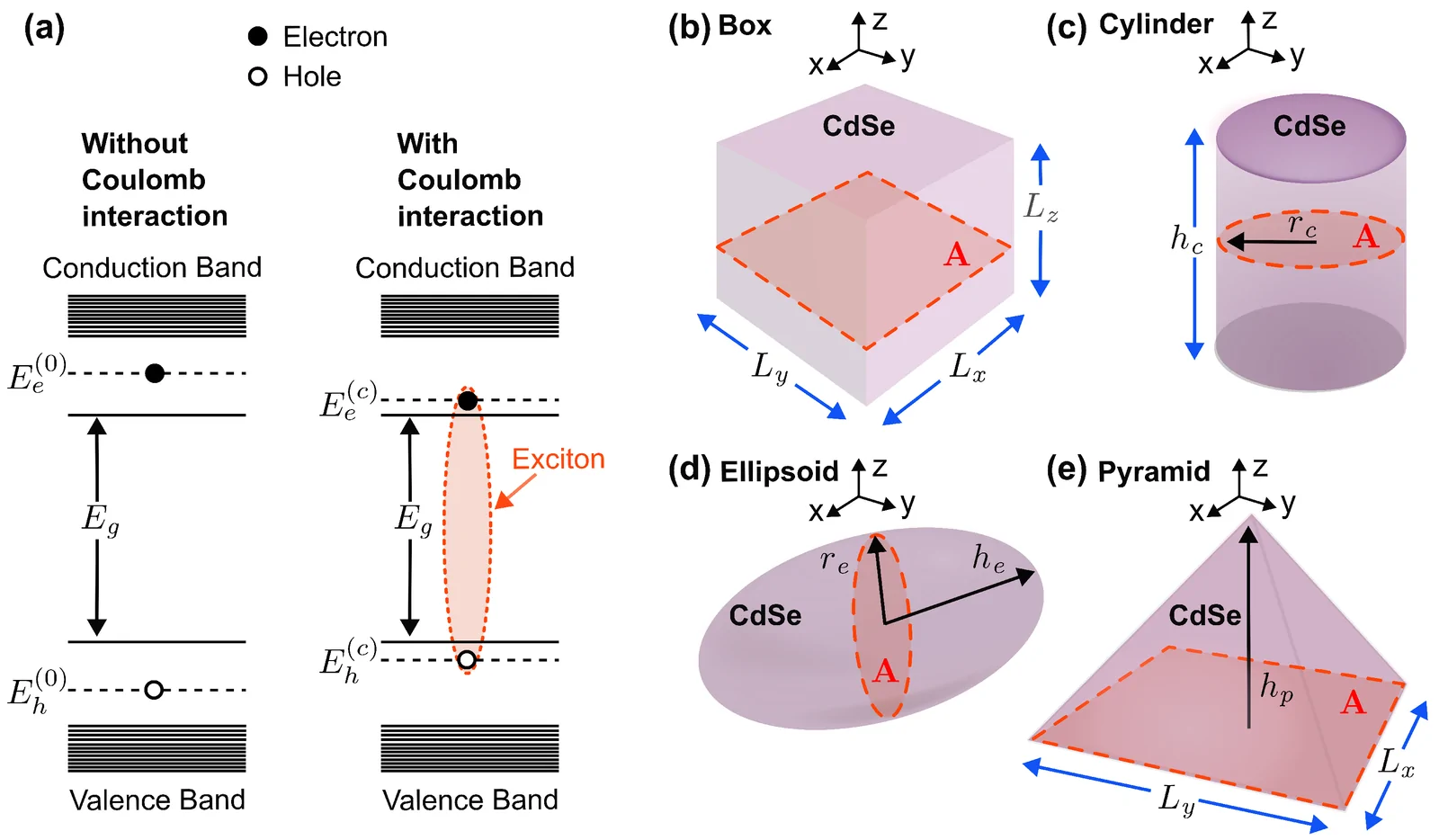
(**a**) Schematic energy-band diagram of the CdSe exciton without (left) and with (right) the effect of the attractive electron–hole Coulomb potential, showing the corresponding electron and hole energies $E_{e}^{\left(0\right)}$, $E_{h}^{\left(0\right)}$ and $E_{e}^{\left(c\right)}$, $E_{h}^{\left(c\right)}$, and the band gap $E_{g}$. (**b**–**e**) Geometry of the four quantum dots studied: box, cylinder, ellipsoid, and square-base pyramid, with their characteristic dimensions. The red dashed contour indicates the cross-sectional area *A* in the xy-plane, taken equal for all structures, as well as the volume *V*.

The exciton is a quasiparticle formed by an interacting electron–hole pair; their mutual Coulomb interaction modifies the potential profiles and the corresponding electron and hole energy levels in the conduction and valence bands, respectively (see Figure 1a). In this work, we aim to calculate the exciton transition energy in QDs with different geometries.

In this study, we have modeled nanoscopic QDs of four shapes: a square-based box ($L_{x}=L_{y}=L$), a cylinder, an ellipsoid of circular cross section, and a square-based pyramid, shown schematically in Figure 1b–e, respectively, together with their characteristic dimensions and coordinate system. To isolate the effect of shape, all structures are constrained to have the same cross-sectional area $A=L^{2}$ and the same volume $V=L^{2}\;L_{z}$, with every dimension expressed through the two parameters *L* and $L_{z}$. The resulting relations are $r_{c}=L/\sqrt{\pi }$ and $h_{c}=L_{z}$ (cylinder); $r_{e}=L/\sqrt{\pi }$ and $h_{e}=\left(3/4\right)\;L_{z}$ (ellipsoid); and $h_{p}=3\;L_{z}$ (pyramid). This allows all four QDs to be varied simultaneously through *L* and $L_{z}$ while preserving equal cross-sectional area and volume [48,50].

Within the effective-mass approximation, the single-exciton Schrödinger equation for a nanoscopic QD reads:

(1) $$\left[-\frac{\hbar^{2}}{2m_{e}^{*}}\nabla_{e}^{2}-\frac{\hbar^{2}}{2m_{h}^{*}}\nabla_{h}^{2}-\frac{e^{2}}{4\pi \;\varepsilon_{0}\;\varepsilon_{r}\left|r_{e}-r_{h}\right|}+V_{e}\left(r_{e}\right)+V_{h}\left(r_{h}\right)\right]\Psi_{\mathrm{exc}}\left(r_{e},r_{h}\right)=E_{\mathrm{exc}}\;\Psi_{\mathrm{exc}}\left(r_{e},r_{h}\right)\;,$$

where *ℏ* is the reduced Planck constant, $m_{e}^{*}$ and $m_{h}^{*}$ are the electron and hole effective masses, taken as constants since the QD consists of a single material (CdSe), *e* is the elementary charge, $\varepsilon_{0}$ is the vacuum permittivity, $\varepsilon_{r}$ is the relative dielectric constant of the medium, $r_{e}$ and $r_{h}$ are the electron and hole position vectors, $\Psi_{\mathrm{exc}}\left(r_{e},r_{h}\right)$ is the exciton wavefunction, and $E_{\mathrm{exc}}$ is the exciton energy eigenvalue. On the left-hand side of Equation (1) the first two terms are the kinetic energies of the electron and hole, the third term is the attractive electron–hole Coulomb interaction, and the last two terms, $V_{e}\left(r_{e}\right)$ and $V_{h}\left(r_{h}\right)$, are the confining potentials, which represent the three-dimensional confinement of the carriers and are given by:

(2) $$V_{e,h}\left(r\right)=\left\{\begin{array}{cc} 0, & r\in \Omega \\ \infty , & r\notin \Omega \;, \end{array}\right.$$

where Ω represents the domain of the QD.

Under the Hartree approximation, the electron moves in a potential created by the hole, and similarly, the hole moves in a potential formed by the electron [38,51,52]. Therefore, Equation (1) can be rewritten as the following pair of coupled single-particle equations:

(3) $$\begin{array}{cc} \left[-\frac{\hbar^{2}}{2m_{e}^{*}}\;\nabla^{2}+V_{e}\left(r\right)-e\;\varsigma_{h}\left(r\right)\right]\psi_{e}\left(r\right)\; & =E_{e}\;\psi_{e}\left(r\right)\;, \end{array}$$

(4) $$\begin{array}{cc} \left[-\frac{\hbar^{2}}{2m_{h}^{*}}\;\nabla^{2}+V_{h}\left(r\right)+e\;\varsigma_{e}\left(r\right)\right]\psi_{h}\left(r\right)\; & =E_{h}\;\psi_{h}\left(r\right)\;, \end{array}$$

subject to the Dirichlet boundary conditions $\psi_{e}\left(r\right){|}_{\partial \Omega }=\psi_{h}\left(r\right){|}_{\partial \Omega }=0$. Here $\varsigma_{e}\left(r\right)$ and $\varsigma_{h}\left(r\right)$ are the Hartree potentials generated by the electron and hole charge densities, respectively, obtained from the Poisson equations,

(5) $$\begin{array}{cc} \nabla^{2}\varsigma_{e}\left(r\right) & =\frac{\rho_{e}\left(r\right)}{\varepsilon_{0}\varepsilon_{r}}=\frac{e\;|\psi_{e}{\left(r\right)|}^{2}}{\varepsilon_{0}\varepsilon_{r}}, \end{array}$$

(6) $$\begin{array}{cc} \nabla^{2}\varsigma_{h}\left(r\right) & =-\frac{\rho_{h}\left(r\right)}{\varepsilon_{0}\varepsilon_{r}}=-\frac{e\;|\psi_{h}{\left(r\right)|}^{2}}{\varepsilon_{0}\varepsilon_{r}}, \end{array}$$

where, for a single exciton, only the lowest electron and hole states are considered. Because $\varsigma_{h}$ depends on $\psi_{h}$ and $\varsigma_{e}$ depends on $\psi_{e}$, Equations (3) and (4) and the Poisson Equations (5) and (6) constitute a coupled system that must be solved self-consistently.

Note that within the present single-band effective-mass approximation, $\psi_{e}$ and $\psi_{h}$ represent the envelope functions of the conduction and valence band-edge states. The microscopic Bloch functions, including the predominantly *s*-like character of the conduction-band edge and *p*-like character of the valence-band edge, are not explicitly resolved. Their different band dispersions are incorporated phenomenologically through the corresponding effective masses.

### 2.2. The Self-Consistent Process

**Figure 2:**
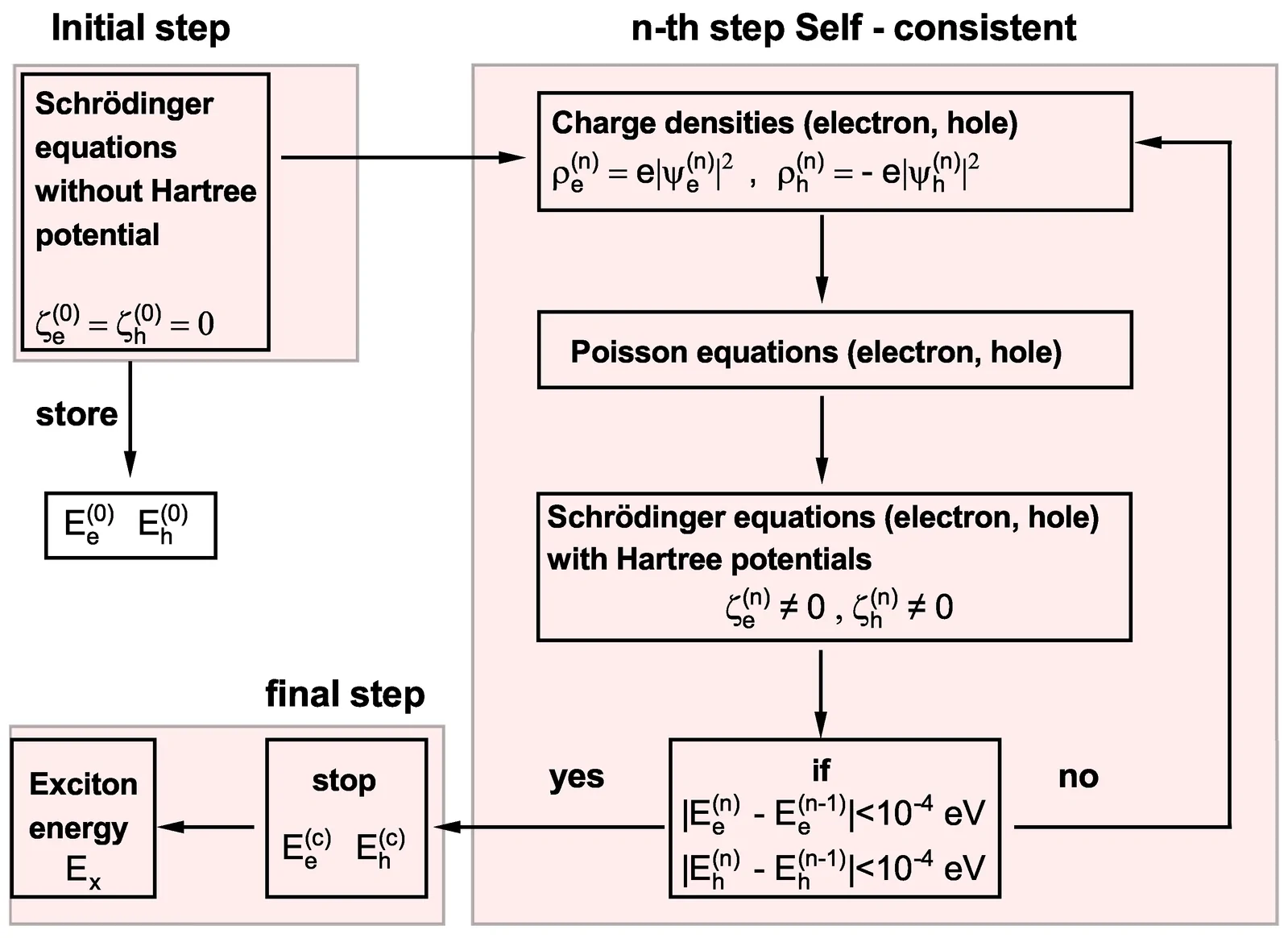
Block diagram of the self-consistent solution of the coupled Schrödinger-Poisson equations for a single exciton confined in a QD.

The coupled Schrödinger–Poisson system defined by Equations (3)–(6) is solved iteratively following the block diagram described in Figure 2. For a given shape and a given pair of parameters $\left(L,\;L_{z}\right)$, the three-dimensional domain Ω is first constructed and discretized with a finite-element mesh in COMSOL Multiphysics 6.1 [53,54,55,56], which is used to solve both the eigenvalue and electrostatic problems.

The self-consistent process begins with the non-interacting problem, so the Hartree potentials are set to zero in the initial step (step 0), $\varsigma_{e}^{\left(0\right)}=\varsigma_{h}^{\left(0\right)}=0$, and Equations (3) and (4) are solved, including only the confinement potential for both the electron and the hole to obtain the Coulomb-free electron and hole energies $E_{e}^{\left(0\right)}$ and $E_{h}^{\left(0\right)}$, together with their corresponding wave functions $\psi_{e}^{\left(0\right)}$ and $\psi_{h}^{\left(0\right)}$. The energies $E_{e}^{\left(0\right)}$ and $E_{h}^{\left(0\right)}$ are stored since they are required for calculating the exciton energy in the final step. In the next step, the resulting wave functions are used to construct the electron and hole charge densities, $\rho_{e}^{\left(0\right)}=e\;{\left|\psi_{e}^{\left(0\right)}\right|}^{2}$ and $\rho_{h}^{\left(0\right)}=e\;{\left|\psi_{h}^{\left(0\right)}\right|}^{2}$, respectively. With these densities, the Poisson Equations (5) and (6) are solved to obtain the Hartree potentials $\varsigma_{e}^{\left(1\right)}$ and $\varsigma_{h}^{\left(1\right)}$, which are, in turn, reinserted into Equations (3) and (4) to yield updated energies and wave functions ($E_{e}^{\left(1\right)}$ and $\psi_{h}^{\left(1\right)}$ in step 1). This sequence is repeated, comparing the electron and hole energies at the *n*-th step with those obtained in the previous step until the electron and hole energies change by less than the prescribed tolerance, $|E_{e,h}^{\left(n\right)}-E_{e,h}^{\left(n-1\right)}|<10^{-4}$ eV. When this condition is satisfied, the iterative process is terminated, and the electron and hole interaction energies $E_{e}^{\left(c\right)}$ and $E_{h}^{\left(c\right)}$ are obtained.

Using the energies obtained in the final self-consistent step ($E_{e}^{\left(c\right)}$ and $E_{h}^{\left(c\right)}$), which include the Coulomb interaction between the electron and hole, together with the noninteracting energies obtained in the initial step ($E_{e}^{\left(0\right)}$ and $E_{h}^{\left(0\right)}$), an expression for the exciton transition energy is obtained:

(7) $$E_{X}=E_{g}+E_{e}^{\left(0\right)}+E_{h}^{\left(0\right)}+E_{eh}^{c}\;,$$

where $E_{g}$ is the band gap energy of the bulk semiconductor material and $E_{eh}^{c}$ is the Coulomb energy between the electron and hole. When calculating the total energy, the Coulomb interaction is already incorporated into the self-consistent electron and hole energies. Consequently, including the Coulomb contribution again would result in double counting. The interaction energy associated with each carrier is given by the shift between its non-interacting and self-consistent values, ($E_{e}^{\left(0\right)}-E_{e}^{\left(c\right)}$) and ($E_{h}^{\left(0\right)}-E_{h}^{\left(c\right)}$) respectively. Averaging these two equivalent measures of the same interaction removes the double counting and yields the singly-counted Coulomb correction, and the factor 1/2 arising directly from this averaging. Considering the above, the term $E_{eh}^{c}$ can be written as:

(8) $$E_{eh}^{c}=-\frac{\left(E_{e}^{\left(0\right)}-E_{e}^{\left(c\right)}+E_{h}^{\left(0\right)}-E_{h}^{\left(c\right)}\right)}{2}\;.$$

From the last two equations, a more compact expression for the exciton transition energy is obtained.

(9) $$E_{X}=E_{g}+\frac{\left(E_{e}^{\left(0\right)}+E_{h}^{\left(0\right)}\right)+\left(E_{e}^{\left(c\right)}+E_{h}^{\left(c\right)}\right)}{2}\;.$$

## 3. Results

As previously mentioned, we implemented the procedure described above in COMSOL Multiphysics using the finite element method. The main characteristics of the mesh employed for the box-shaped quantum dot are summarized below (for the other QD geometries, these parameters may vary slightly): the mesh consists of 186,273 vertices, 1,015,162 tetrahedral elements, 19,846 triangular elements, 420 edge elements, and 8 vertex elements. The minimum element quality is 0.07102, the average element quality is 0.5768, and the element volume ratio is 0.01456. Since the QDs are composed of CdSe and both the conduction and valence bands are considered, parameters for both electrons and holes are required. Table 1 summarizes the parameters used in the calculations.

The band-gap energy $E_{g}=1.75$ eV corresponds to bulk CdSe and is kept constant throughout the calculations. Within the effective-mass framework adopted here, the size and geometry-dependent increase of the optical transition energy is explicitly accounted for through the electron and hole confinement energies; therefore, no additional size-dependent correction is introduced into $E_{g}$.

**Figure 3:**
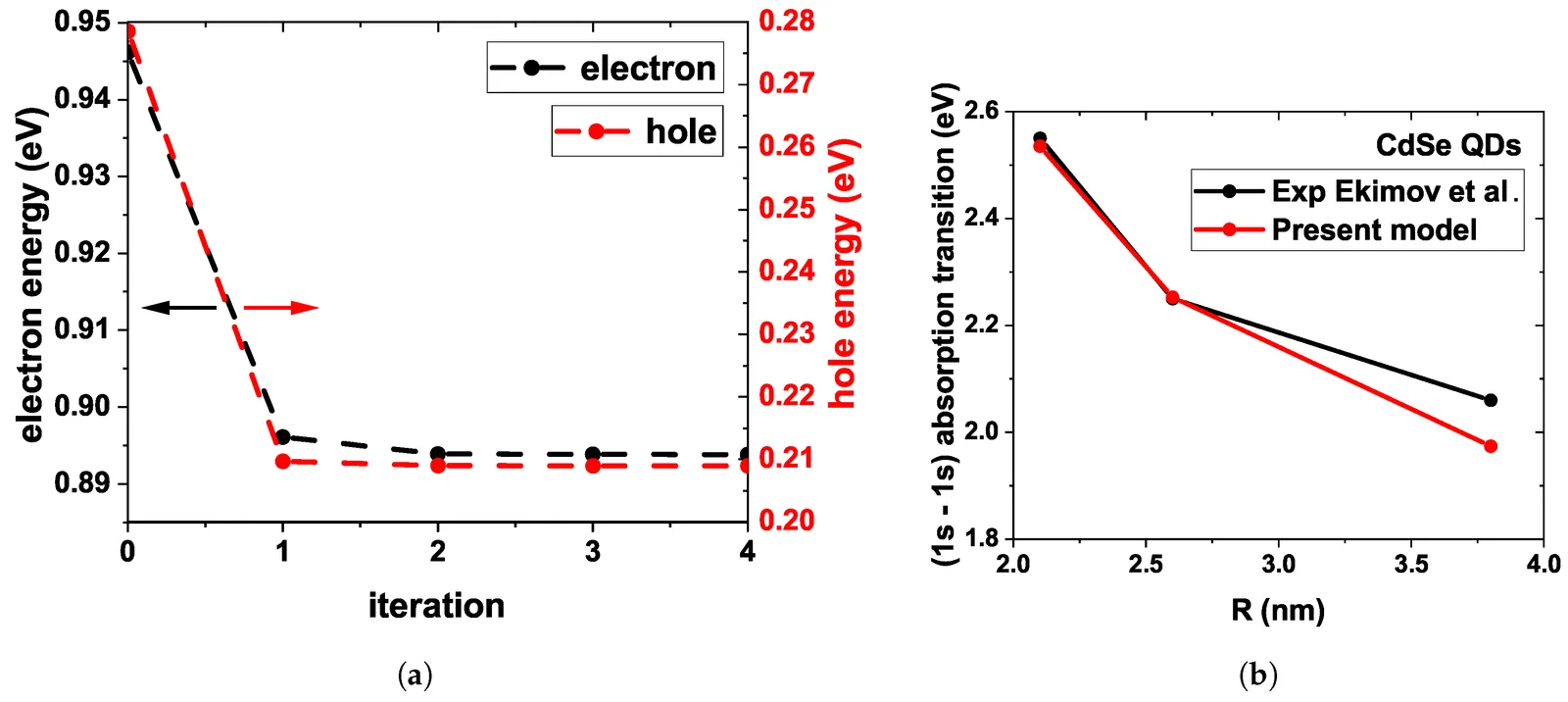
(**a**): Electron and hole energies as a function of the number of iterations in the self-consistent procedure, represented by the black and red symbols, respectively. The results correspond to a 3 nm-edge CdSe cubic QD. (**b**): Comparison between the theoretical results (absorption transition 1s−1s) obtained in this work and the experimental results reported by Ekimov et al. [49] for spherical CdSe QDs.

Figure 3a shows the electron energy (black dots, left axis) and hole energy (red dots, right axis) for a cubic quantum dot with a 3 nm edge length as a function of the number of iterations of the self-consistent procedure described in the previous section. The first point corresponds to the energy without Coulomb interaction (iteration 0), i.e., considering the electron and hole as isolated particles. Starting from the first iteration, the Hartree potential is included, accounting for the interaction between the electron and hole. Clear, rapid convergence is observed, occurring practically by the third or fourth iteration. This provides evidence that the self-consistent algorithm described above yields well-defined converged values for the electron–hole interaction energies.

The theoretical procedure described above is consistent with experimental results. To demonstrate this agreement, Figure 3b presents a comparison between the results obtained using the present approach and the experimental results reported by Ekimov et al. [49] for spherical CdSe QDs with three different radii. In particular, the excitonic transition energy (1s−1s), calculated from Equation (9), is compared with the corresponding absorption data. Very good agreement is observed for QDs with radii of 2.1 nm and 2.6 nm, whereas a somewhat larger deviation is observed for the QD with a radius of 3.8 nm. This can be attributed to the fact that, for small radii (R=2.1 nm and 2.6 nm), the QD is in the strong-confinement regime, where the exciton Bohr radius in CdSe is much larger than the QD radius. At this scale, the confinement kinetic energy dominates over the Coulomb interaction. Therefore, although the model neglects instantaneous spatial correlations between the two particles, it agrees well with experimental results. On the other hand, as the QD size increases, the confinement kinetic energy decreases significantly. The system then enters an intermediate confinement regime, in which the Coulomb attraction and electron-hole spatial correlations become increasingly important. Since explicit particle correlations are not included, the Hartree approximation progressively deviates from the experimental results as *R* increases (as evidenced in Figure 3b).

A quantitative comparison of the calculated and experimental transition energies is summarized in Table 2, the experimental and theoretical values of the exciton energy ($E_{X}^{exp}$ and $E_{X}^{theory}$), the energy deviation $\Delta E=|E_{X}^{theory}-E_{X}^{exp}|$, and the corresponding percentage relative error with respect to the experimental value $error\;\left(\%\right)=\Delta E/E_{X}^{exp}$ are presented. The agreement is better for the smaller QDs, consistent with the strong-confinement regime in which the carrier confinement energy dominates over the electron-hole interaction. As the radius increases, the relative importance of electron-hole correlations increases, while the Hartree approximation accounts for the interaction only at the mean-field level. This provides one possible explanation for the increasing deviation observed for the largest QD. Additional contributions may arise from effects neglected in the present model, including dielectric mismatch between the QD and its environment, band non-parabolicity, and surface/interface effects. Accordingly, the present comparison indicates that the model is quantitatively more reliable in the strong-confinement regime, whereas its predictions for larger structures should be interpreted primarily in terms of relative size and geometry-dependent trends.

**Figure 4:**
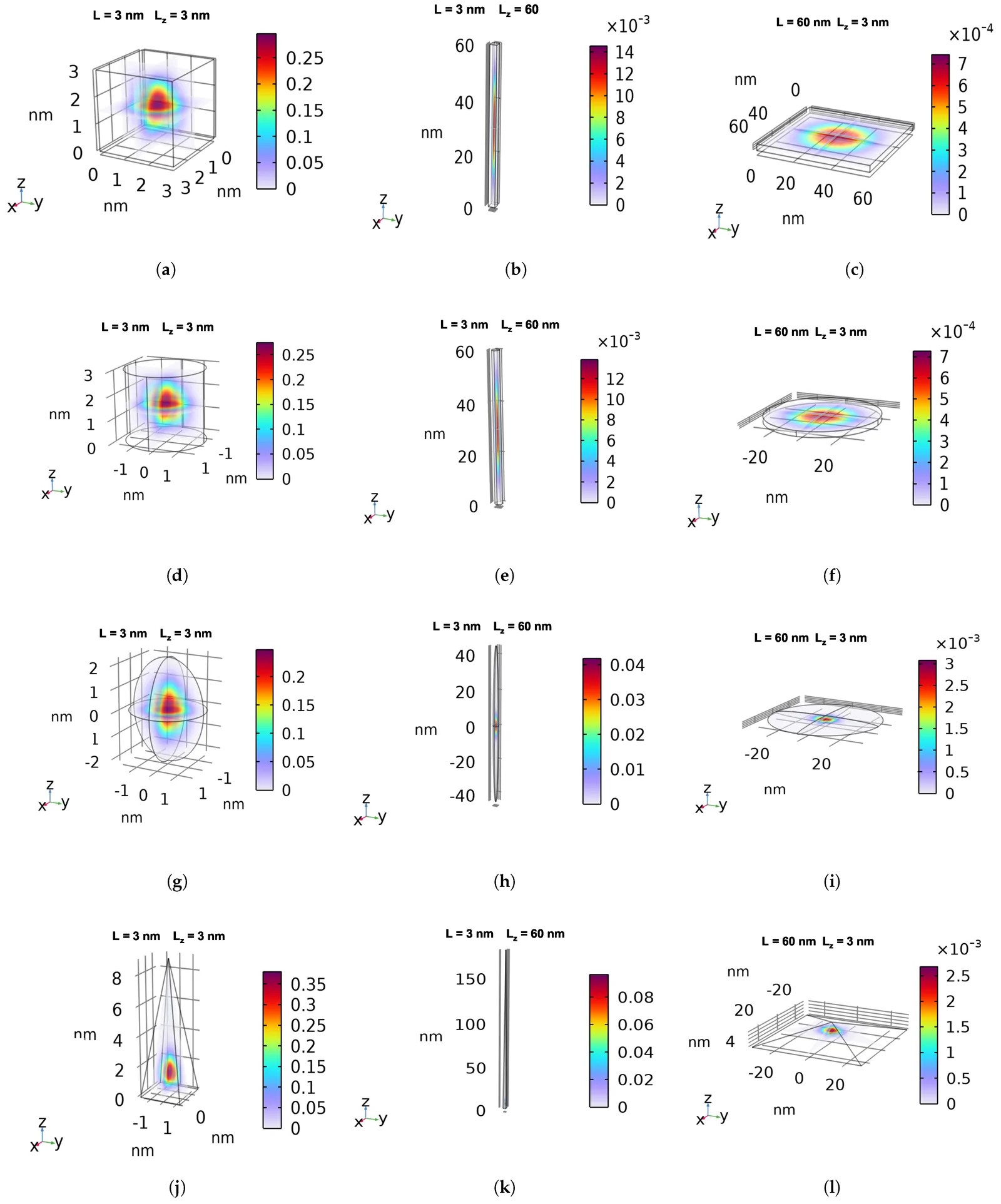
Electron probability density for the four quantum dots studied under three characteristic limiting geometries: $L_{z}=3$ nm, L=3 nm (quantum dot system), $L_{z}=60$ nm, L=3 nm (quantum wire system), and $L_{z}=3$ nm, L=60 nm (two-dimensional quantum system). Figures (**a**–**c**) box, (**d**–**f**) cylinder, (**g**–**i**) ellipsoid, and (**j**–**l**) pyramid.

To visualize the influence of spatial geometry and aspect ratio on the electronic distribution, Figure 4 displays the electron probability density $|\psi_{e}{\left(r\right)|}^{2}$ across the four QD geometries under three characteristic limiting regimes: a zero-dimensional (0D) quantum dot system (L=3 nm, $L_{z}=3$ nm), a quasi-one-dimensional (1D) quantum wire limit (L=3 nm, $L_{z}=60$ nm), and a quasi-two-dimensional (2D) quantum well/nanoplatelet limit (L=60 nm, $L_{z}=3$ nm). In the 0D regime ($L=L_{z}=3$ nm), strong three-dimensional quantum confinement dominates. The electron wave function is highly localized, and its spatial profile closely reflects the domain boundary conditions of each geometry. For symmetric structures (box, cylinder, and ellipsoid), the probability density reaches its maximum at the geometric center. However, in the pyramidal QD, the broken vertical symmetry forces the probability to shift downwards toward the broader base. This displacement occurs because the larger cross-sectional area near the base reduces the local kinetic confinement energy compared to the restricted volume near the apex. When the vertical dimension is extended to $L_{z}=60$ nm while keeping L=3 nm (1D regime), longitudinal quantum confinement along the *z*-axis is substantially decreased. Consequently, the wave function elongates into a cylindrical/rod-like channel while retaining strict lateral confinement in the xy-plane, characteristic of a quantum wire. It should be noted that one consequence of the geometry of each QD is that the probability density corresponding to the ellipsoidal and pyramidal geometries are less spatially extended than those of the box-shaped and cylindrical QDs. Conversely, in the 2D regime (L=60 nm, $L_{z}=3$ nm), strong confinement is maintained exclusively along the vertical axis, allowing the wave function to spread laterally over the xy-plane in a nanoplatelet-like distribution. These dimensional crossovers illustrate how modifying the aspect ratio ($L_{z}/L$) directly tunes the spatial charge density, laying the groundwork for understanding the geometry-dependent Coulomb interaction discussed in subsequent sections.

**Figure 5:**
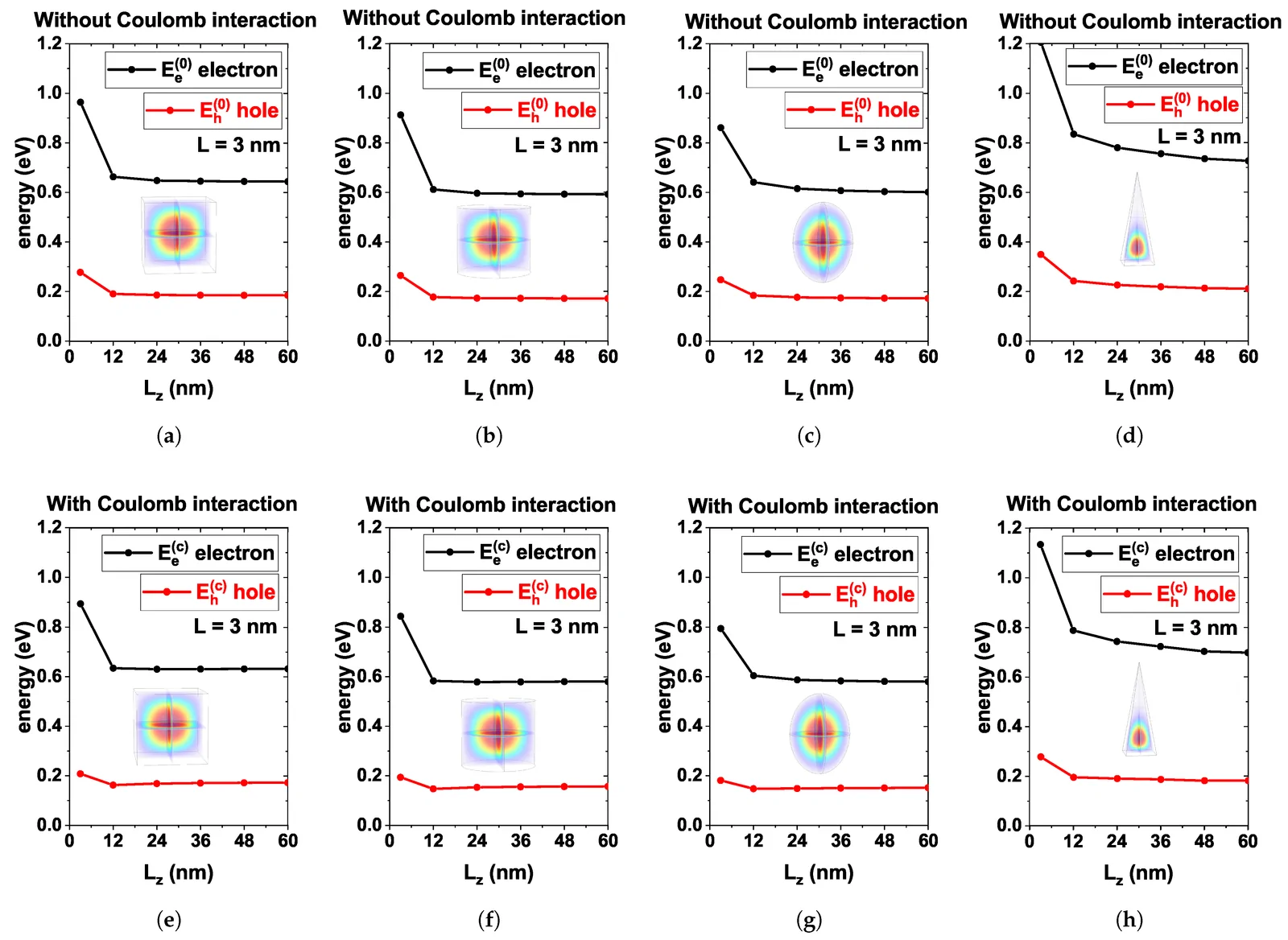
(**a**–**d**): Electron and hole energy without Coulomb interaction for the four QDs studied as a function of the length $L_{z}$, varying from 3 nm to 60 nm, the terms $E_{e}^{\left(0\right)}$ and $E_{h}^{\left(0\right)}$ correspond to the electron and hole energies without Coulomb interaction (black and red curves, respectively). (**e**–**h**): Same as panels (**a**–**d**), but including the Coulomb interaction (self-consistent solution), the terms $E_{e}^{\left(c\right)}$ and $E_{h}^{\left(c\right)}$ correspond to the electron and hole energies with Coulomb interaction (black and red curves, respectively). For all panels, the parameter *L* was fixed at L=3 nm, while keeping both the cross-sectional area (base area for the pyramid) and the volume fixed and equal for the four QDs. To visualize the structure, the ground-state wave function of the electron in the minimum-volume system has been included.

Figure 5 presents the ground-state single-particle energies for the electron ($E_{e}$) and hole ($E_{h}$) as a function of the longitudinal dimension $L_{z}$, varied from 3 nm to 60 nm, while maintaining the transverse parameter fixed at L=3 nm. Panels Figure 5a–d display the non-interacting system ($E_{e}^{\left(0\right)}$ and $E_{h}^{\left(0\right)}$), whereas panels Figure 5e–h show the self-consistent results ($E_{e}^{\left(c\right)}$ and $E_{h}^{\left(c\right)}$) incorporating the attractive Coulomb interaction via the Hartree potential. This variation represents a continuous transition from an isotropic 0D quantum dot ($L_{z}=3$ nm) toward an elongated quasi-1D quantum wire regime ($L_{z}=60$ nm). Across all four geometries: box (a), cylinder (b), ellipsoid (c), and pyramid (d), both $E_{e}^{\left(0\right)}$ and $E_{h}^{\left(0\right)}$ exhibit a sharp, monotonic decrease as $L_{z}$ increases from 3 nm to 60 nm. This behavior reflects the progressive reduction of spatial confinement along the *z*-axis. As $L_{z}\gg L$, the vertical kinetic confinement becomes negligible, and the energy is predominantly determined by the fixed two-dimensional transverse confinement defined by L=3 nm. Although all QDs have the same volume, the pyramidal geometry imposes stronger confinement on both the electron and the hole, as evidenced by the electron wave function shown for each geometry. This enhanced confinement explains why the energy associated with the pyramidal QD is higher than that of the other geometries considered. In all panels, the electron energy ($E_{e}^{\left(0\right)}$) is significantly higher than the hole energy ($E_{h}^{\left(0\right)}$). This difference directly stems from the effective mass ratio ($m_{e}^{*}=0.13\;m_{0}$ vs. $m_{h}^{*}=0.45\;m_{0}$), as kinetic confinement energy scales inversely with effective mass ($E\propto 1/m^{*}$).

The effect of the Coulomb attraction is presented in panels Figure 5e–h, which show the self-consistent energies $E_{e}^{\left(c\right)}$ and $E_{h}^{\left(c\right)}$. Comparison of these panels with Figure 5a–d reveals a systematic downward shift in the energy of both carriers across the entire range of $L_{z}$. This shift reflects the attractive mean-field potential each carrier experiences due to the charge distribution of the other carrier. In the 0D regime ($L_{z}=3$ nm), strong spatial overlap between electron and hole wave functions leads to maximum Coulomb attraction, causing the largest drop from $E^{\left(0\right)}$ to $E^{\left(c\right)}$. As $L_{z}$ increases, the wave functions spread along the *z*-axis, slightly reducing the local charge density and weakening the attractive Hartree potential. Consequently, the gap between $E^{\left(0\right)}$ and $E^{\left(c\right)}$ diminishes gradually as the system approaches the 1D quantum wire limit.

**Figure 6:**
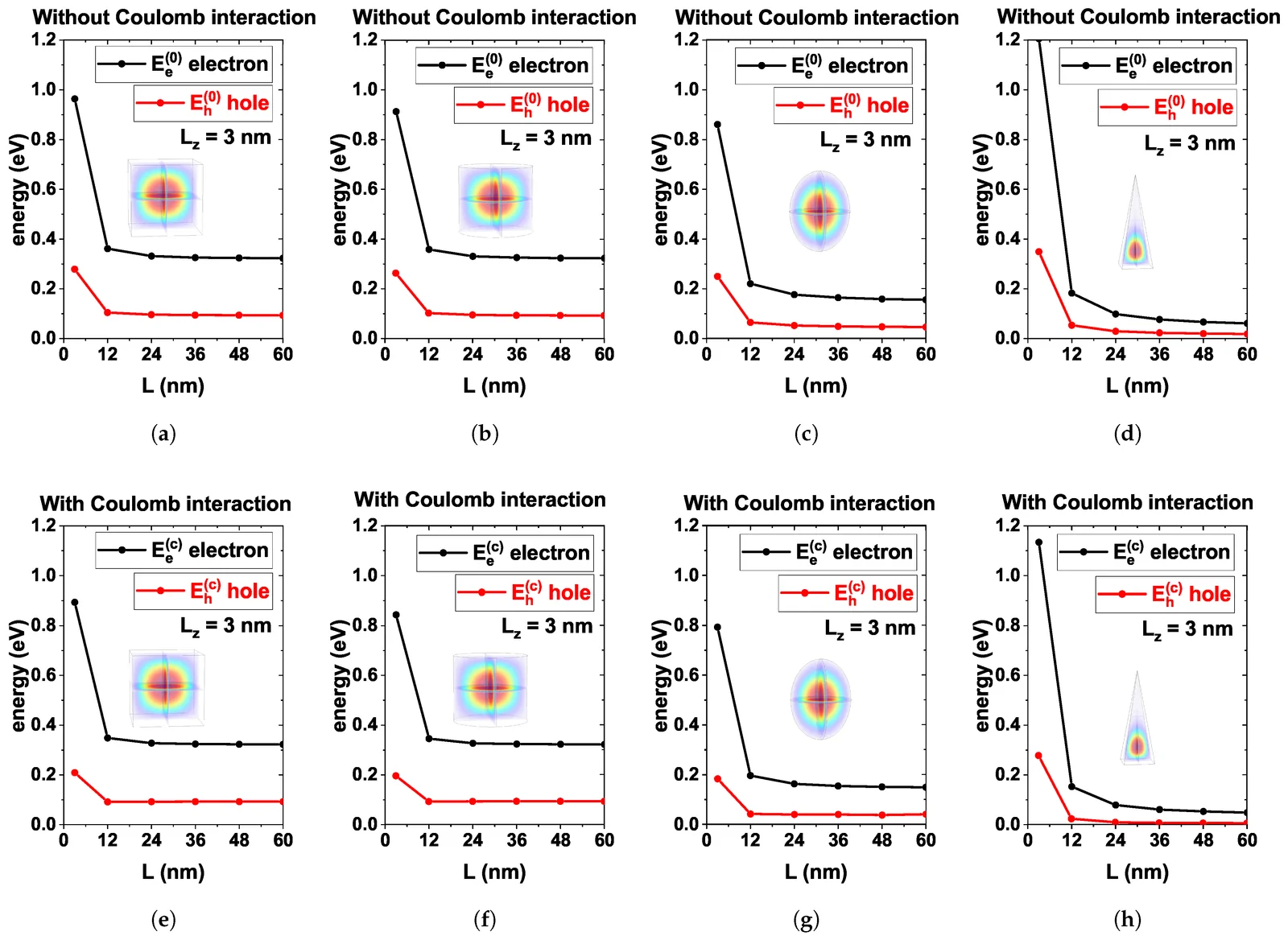
(**a**–**d**): Electron and hole energy without Coulomb interaction for the four QDs studied as a function of the length *L*, varying from 3 nm to 60 nm, the terms $E_{e}^{\left(0\right)}$ and $E_{h}^{\left(0\right)}$ correspond to the electron and hole energies without Coulomb interaction (black and red curves, respectively). (**e**–**h**): Same as panels (**a**–**d**), but including the Coulomb interaction (self-consistent solution), the terms $E_{e}^{\left(c\right)}$ and $E_{h}^{\left(c\right)}$ correspond to the electron and hole energies with Coulomb interaction (black and red curves, respectively). For all panels, the parameter $L_{z}$ was fixed at $L_{z}=3$ nm, while keeping both the cross-sectional area (base area for the pyramid) and the volume fixed and equal for the four QDs. To visualize the structure, the ground-state wave function of the electron in the minimum-volume system has been included.

Figure 6 illustrates the ground-state single-particle energies for the electron ($E_{e}$) and hole ($E_{h}$) as a function of the transverse dimension *L*, varied from 3 nm to 60 nm, while holding the longitudinal parameter fixed at $L_{z}=3$ nm. Panels Figure 6a–d show the non-interacting carrier energies ($E_{e}^{\left(0\right)}$ and $E_{h}^{\left(0\right)}$), whereas panels Figure 6e–h correspond to the self-consistent interacting energies ($E_{e}^{\left(c\right)}$ and $E_{h}^{\left(c\right)}$) obtained after incorporating the attractive Hartree potentials. In contrast to Figure 5, varying *L* expands both transverse dimensions (*x* and *y*) simultaneously, driving a continuous dimensional crossover from an isotropic 0D quantum dot ($L=L_{z}=3$ nm) to a quasi-two-dimensional (2D) quantum well or nanoplatelet regime (L=60 nm, $L_{z}=3$ nm). Across all four geometries: box (a), cylinder (b), ellipsoid (c), and pyramid (d), both $E_{e}^{\left(0\right)}$ and $E_{h}^{\left(0\right)}$ exhibit a monotonic decrease over the entire range of *L*. This initial drop is notably sharper than that observed in Figure 5 when varying $L_{z}$. The difference arises because increasing *L* expands the cross-sectional area quadratically ($A=L^{2}$), decreasing spatial kinetic confinement along two orthogonal directions (*x* and *y*) simultaneously. As *L* expands beyond 25nm ($L\gg L_{z}$), lateral kinetic energy contributions ($\propto 1/L^{2}$) vanish, and the carrier energies asymptote toward constant plateaus. In this limit, the system behaves as a 2D quantum well where the kinetic energy is governed exclusively by the vertical confinement imposed by $L_{z}=3\;\mathrm{nm}$. Again, in all panels, $E_{e}^{\left(0\right)}$ remains substantially higher than $E_{h}^{\left(0\right)}$ due to the effective mass disparity, reaffirming that the lighter electron experiences stronger quantum confinement kinetic energy ($E\propto 1/m^{*}$) than the hole. The Box (a) and Cylinder (b) display nearly identical decay curves and asymptotic plateaus because both structures maintain uniform vertical cross-sections and equal vertical heights ($h_{c}=L_{z}=3$ nm).

Note that the ellipsoid has the same cross-sectional area as the cylinder, with its major semi-axis given by $r_{e}=3L_{z}/4$. Therefore, the height of the ellipsoid along the *z*-axis is twice the major semi-axis, $2\left(3L_{z}/4\right)=3L_{z}/2$. Consequently, the ellipsoid has a greater height than both the cylinder and the box. This reduction in confinement along the *z*-direction leads to a decrease in the ground-state energy of both the electron and the hole compared with the two aforementioned systems, as evidenced in Figure 6c. The pyramid shows the lowest asymptotic energy in the large-*L* limit. Because its height scales as $h_{p}=3L_{z}=9$ nm, extending *L* produces a much taller structure than the other geometries, substantially decreasing the vertical confinement. In Figure 6e–h incorporate the Hartree potentials, resulting in a uniform downward shift of both the electron and hole energy levels, such that $E_{e}^{\left(c\right)}<E_{e}^{\left(0\right)}$ and $E_{h}^{\left(c\right)}<E_{h}^{\left(0\right)}$ across all values of *L*. At L=3 nm, strong 3D spatial overlap yields a large Coulomb binding energy. As *L* increases, the electron and hole wave functions spread out across the xy-plane (as illustrated in Figure 4 for L=60 nm), diluting the in-plane charge density ρ(r). This 2D lateral spreading weakens the attractive mean-field Hartree potential, causing the gap between $E^{\left(0\right)}$ and $E^{\left(c\right)}$ to narrow as *L* approaches 60 nm. However, because vertical confinement remains tight ($L_{z}=3$ nm), the carriers remain localized along *z*, preserving a finite Coulomb attraction even in the 2D nanoplatelet limit.

**Figure 7:**
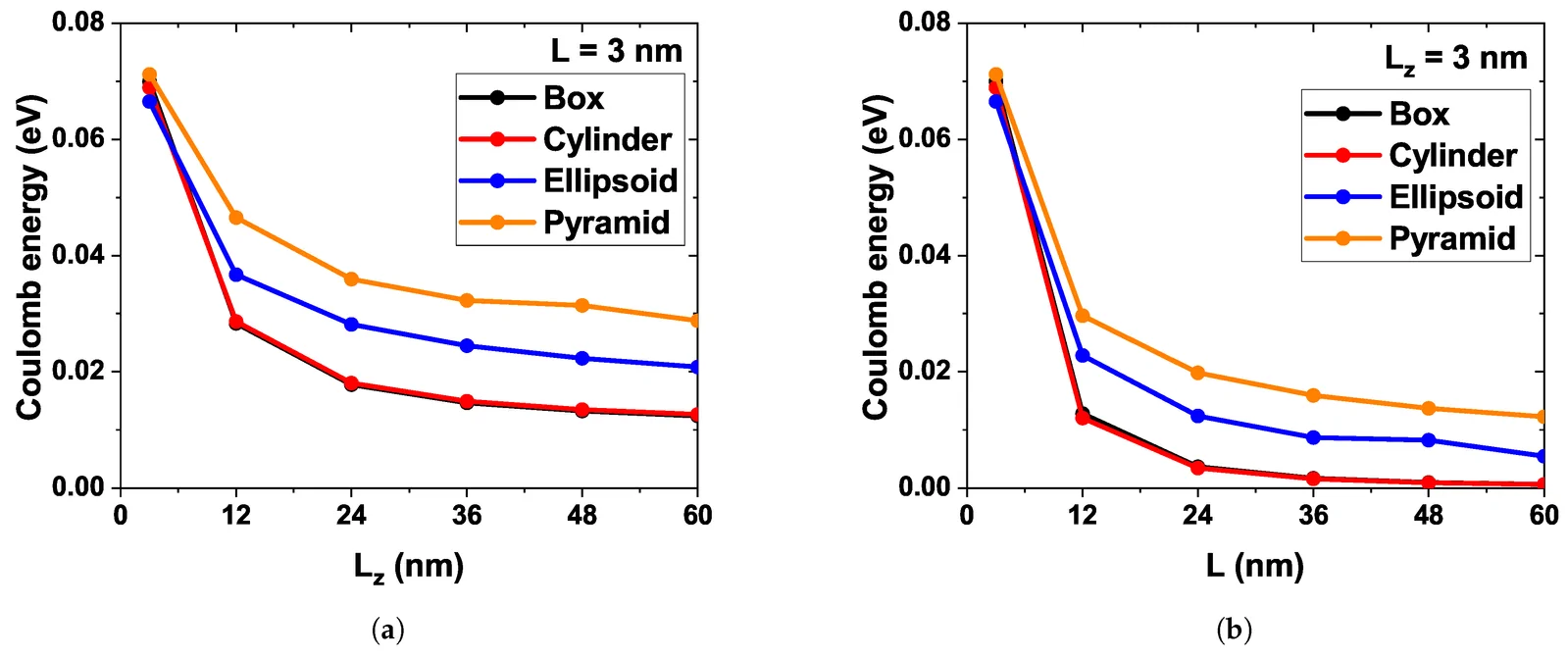
Coulomb energy for the four QDs studied as a function of $L_{z}$, with L=3 nm fixed (panel (**a**)), and as a function of *L*, with $L_{z}=3$ nm fixed (panel (**b**)). For each variation, all QDs have the same volume and the same cross-sectional area (base area in the case of the pyramidal QD).

Figure 7 provides a direct measure of the effect of the electron–hole Coulomb interaction on the four QD geometries by showing the magnitude of the Coulomb energy as a function of the longitudinal dimension ($L_{z}$) and the transverse dimension (*L*). This result complements the single-particle energy analysis presented in Figure 5 and Figure 6, since the differences observed between the non-interacting and self-consistent energies are ultimately determined by the spatial overlap and localization of the electron and hole wave functions. In the following discussion, the positive quantity represented in Figure 7 is understood as the magnitude of the Coulomb contribution ($\left|E_{eh}^{c}\right|$), so that larger values correspond to stronger electron–hole attraction. For the variation of $L_{z}$ at fixed L=3 nm (Figure 7a), all four structures exhibit a pronounced decrease in the Coulomb energy as the longitudinal dimension increases. At $L_{z}=3$ nm, the Coulomb energies are very similar for the four geometries, with values close to 0.07 eV. This behavior is consistent with the strong-confinement regime discussed previously, where the relatively small dimensions of the structures force both carriers into a highly localized region and produce a substantial spatial overlap between their probability densities. Consequently, the details of the external shape have only a moderate influence on the Coulomb interaction when the QDs are small in all three spatial directions. The situation changes significantly as $L_{z}$ increases. The Coulomb energy decreases rapidly between $L_{z}=3$ nm and approximately 12 nm, followed by a progressively slighter decrease at larger values of $L_{z}$. This behavior can be understood from the spatial evolution of the carrier wave functions shown in Figure 4. Increasing $L_{z}$ reduces the confinement along the *z*-direction, allowing both the electron and hole densities to spread over a larger region. The resulting decrease in local charge density reduces the average electron-hole attraction and, consequently, the magnitude of the Coulomb binding energy. Thus, the strong decrease observed in Figure 7a is a direct consequence of the dimensional crossover from a strongly confined zero-dimensional structure toward a quasi-one-dimensional regime.

An interesting geometric dependence emerges in the large-$L_{z}$ regime. The box and cylinder exhibit nearly indistinguishable Coulomb energies over the entire range, whereas the ellipsoid maintains a larger Coulomb contribution and the pyramid displays the largest value. At $L_{z}=60$ nm, for example, the Coulomb energy is approximately 0.012 eV for the box and cylinder, 0.021 eV for the ellipsoid, and 0.029 eV for the pyramid. The similarity between the box and cylinder is expected because both structures have the same transverse area and the same longitudinal height, so their confinement along the three relevant directions is comparable in the present configuration. The ellipsoid, in contrast, has a larger extent along *z*, which modifies the spatial distribution of the carriers and produces a geometry-dependent balance between longitudinal delocalization and transverse confinement. The pyramid shows the largest Coulomb energy despite its extended height. This result is associated with its nonuniform cross section: the available volume decreases continuously toward the apex, favoring a stronger localization of the ground-state wave functions toward the broader base, as observed in Figure 4. The resulting carrier densities remain more concentrated in a common spatial region, enhancing their effective overlap and preserving stronger electron-hole attraction.

Figure 7b reveals a more pronounced reduction of the Coulomb energy when the transverse dimension *L* is increased while $L_{z}=3$ nm is kept fixed. In the strongly confined configuration at L=3 nm, the four structures again present Coulomb energies close to 0.07 eV. However, increasing *L* produces a much faster decrease than that observed when varying $L_{z}$. This difference is expected because *L* controls both transverse dimensions simultaneously (xy). Therefore, increasing *L* enlarges the confinement region in the *x* and *y* directions at the same time, producing a more substantial reduction in carrier density than an equivalent increase of $L_{z}$, where only the confinement along one direction is relaxed. As *L* increases beyond approximately 20–25 nm, the Coulomb energy of the different structures approaches a much smaller value, particularly for the box and cylinder. In these systems, the electron and hole wave functions become increasingly extended in the xy-plane, while the fixed value $L_{z}=3$ nm maintains strong confinement along *z*. This reduction in in-plane charge density substantially weakens the mean-field Coulomb interaction. Nevertheless, the Coulomb energy does not vanish completely. The finite confinement in the *z* direction continues to localize the carriers vertically, maintaining a nonzero electron-hole overlap even in the quasi-two-dimensional limit. This behavior is consistent with the spatial distributions shown in Figure 4 for L=60 nm.

The geometrical differences in Figure 7b are particularly evident at large *L*. The box and cylinder exhibit the weakest Coulomb interaction, while the ellipsoid and, especially, the pyramid retain significantly larger Coulomb energies. At L=60 nm, the Coulomb contribution is close to zero for the box and cylinder, whereas it remains around 0.005 eV for the ellipsoid and approximately 0.012 eV for the pyramid. This ordering is consistent with the different spatial distributions imposed by the geometries. For the box and cylinder, expanding the transverse dimensions produces an almost unrestricted lateral spreading of the carriers. In the ellipsoid, the continuously varying boundary modifies this spreading and leads to a somewhat stronger spatial localization. The pyramid produces the strongest effect because its nonuniform cross section prevents the charge density from becoming uniformly distributed throughout the enlarged lateral region. Instead, the ground-state wave functions remain preferentially localized toward the wider portion of the structure, allowing a larger spatial overlap between the electron and hole.

The comparison between the panels of Figure 7a,b therefore demonstrates that the Coulomb interaction is not determined solely by the total volume of the QD. Although the four structures are constructed with identical volume and cross-sectional area for each pair of *L* and $L_{z}$, their Coulomb energies can differ considerably, particularly as the structures approach the quasi-1D or quasi-2D limits. The relevant quantity is the spatial distribution of the electron and hole densities within that volume. In other words, two QDs with the same volume can exhibit different excitonic properties because their boundaries impose different localization conditions on the carriers. These results also clarify the behavior observed previously in Figure 5 and Figure 6. The downward displacement of the self-consistent electron and hole energies relative to their non-interacting values is largest when the carriers are strongly confined, and their wave functions substantially overlap. As either $L_{z}$ or *L* increases, the reduction of the Coulomb energy explains the progressive narrowing of the separation between $E_{e,h}^{\left(0\right)}$ and $E_{e,h}^{\left(c\right)}$. The stronger Coulomb contribution retained by the ellipsoidal and pyramidal structures therefore indicates that their geometric confinement continues to influence the exciton even when the system approaches a lower-dimensional limit.

**Figure 8:**
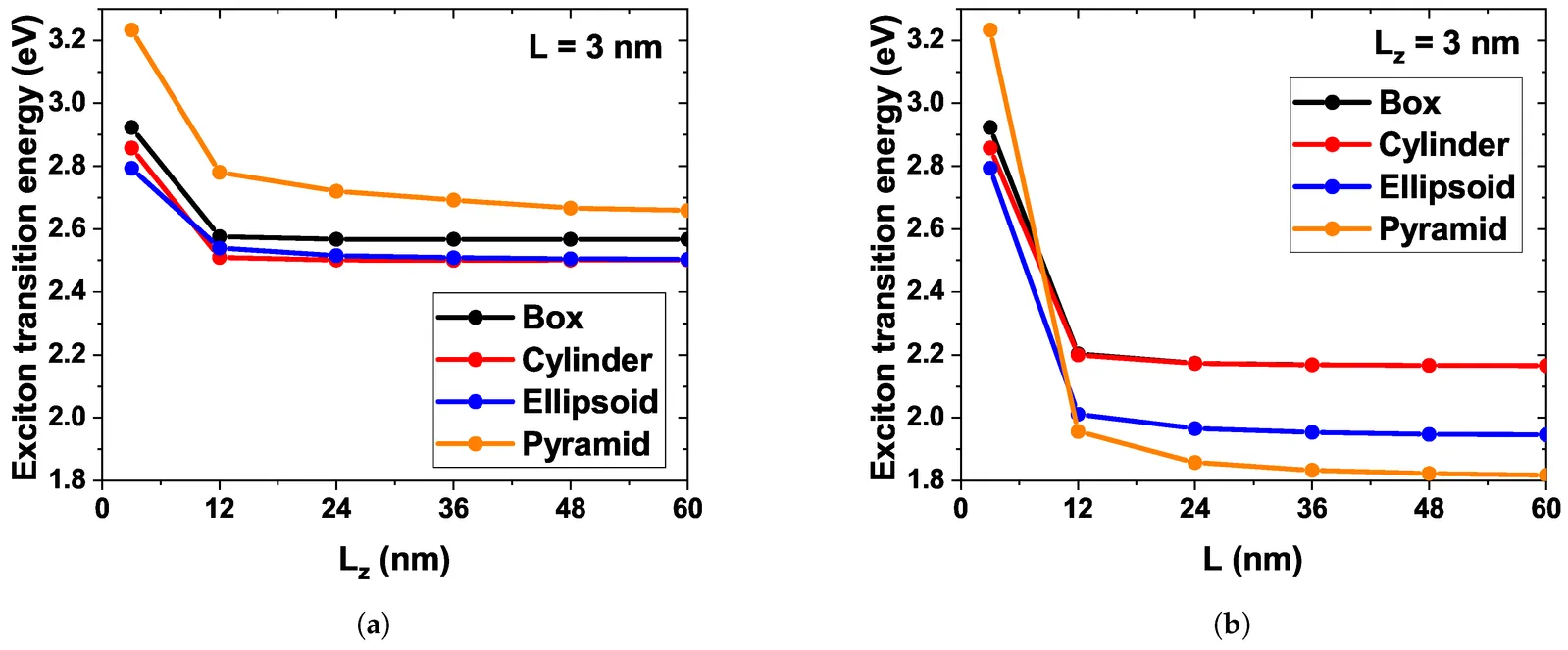
Exciton transition energy for the four QDs studied, considering variations in the parameters $L_{z}$ and *L* (panels (**a**,**b**), respectively). For each variation, all QDs have the same volume and the same cross-sectional area (base area in the case of the pyramidal QD). Both panels use the same scale to facilitate comparison.

Overall, Figure 7 shows that the electron–hole Coulomb interaction is highly sensitive to the aspect ratio and boundary geometry of the QD. The strongest Coulomb binding occurs for the smallest structures, where the electron and hole are strongly localized, whereas increasing either dimension progressively weakens their interaction. More importantly, the results show that geometry can preserve a substantial Coulomb contribution even when one dimension becomes comparatively large. This geometry-dependent Coulomb binding is expected to be reflected directly in the exciton transition energies presented in Figure 8, where the effects of quantum confinement and electron-hole attraction are combined.

To characterize the dimensional crossovers quantitatively, we introduce the aspect ratios $\eta_{1D}=L_{z}/L$ and $\eta_{2D}=L/L_{z}$, which measure the extension toward the quantum-wire and nanoplatelet regimes, respectively. Taking the bulk CdSe exciton Bohr radius ($a_{B}\approx 5.6$ nm) as the reference scale [60], the fixed 3 nm dimension used in both sweeps lies well within the strong-confinement limit, so that the growing dimension governs the crossovers. In the longitudinal sweep (L=3 nm fixed), the carrier and Coulomb energies vary strongly for $L_{z}≲12$ nm and approach a plateau controlled by the fixed transverse confinement beyond that value. We therefore define $n_{1D}≳4$ as the operational onset of the quasi-1D regime. In the transverse sweep ($L_{z}=3$ nm fixed), the energies become nearly invariant for L≳20−25 nm, corresponding to $n_{2D}\approx 7-8$. The larger aspect ratio required in the latter case reflects the fact that increasing *L* relaxes confinement along two orthogonal directions simultaneously ($A=L^{2}$), whereas increasing $L_{z}$ relaxes confinement along a single direction. The above thresholds should be understood as model-specific crossover criteria rather than universal dimensional. The following Table 3 summarizes the above geometrical criteria.

Figure 8 summarizes the combined effects of quantum confinement and electron-hole Coulomb attraction on the exciton transition energy of the four QD geometries. In both panels, the transition energy decreases as the characteristic QD size increases, indicating that the reduction in quantum confinement dominates over the simultaneous weakening of the Coulomb interaction discussed in Figure 7. Thus, increasing the dimensions of the structures produces a progressive redshift of the excitonic transition. For the variation of $L_{z}$ at fixed L=3 nm (Figure 8a), the transition energy decreases monotonically as $L_{z}$ increases from 3 to 60 nm. The pronounced decrease at small $L_{z}$ reflects the rapid reduction of the longitudinal confinement energy, while the progressively weaker variation at larger $L_{z}$ indicates an approach to the quasi-one-dimensional regime. In this limit, the confinement along *z* becomes less relevant, and the transition energy is mainly determined by the fixed transverse confinement.

Among the four geometries, the cylinder and ellipsoid exhibit very similar transition energies over most of the $L_{z}$ range, whereas the box remains slightly above them. This similarity primarily originates from the fact that, as $L_{z}$ increases, the longitudinal confinement becomes progressively less relevant and the transition energy is increasingly governed by the transverse confinement. Since the cylinder and ellipsoid have the same circular cross-sectional area, their dominant transverse confinement contributions become very similar, causing their transition energies to approach each other. As $L_{z}$ increases, the energy contribution associated with the transverse xy-plane becomes increasingly relevant. Since the cylinder and the ellipsoid have exactly the same circular cross-sectional area, their exciton transition energies are expected to converge as $L_{z}$ increases. In contrast, the pyramid consistently shows the highest transition energy, reflecting stronger effective confinement due to its nonuniform cross section. Although the pyramid also exhibits the largest Coulomb contribution, as shown in Figure 7a, this additional electron-hole attraction is not sufficient to compensate for its stronger confinement, resulting in the highest exciton transition energy.

A different behavior is obtained when *L* is varied while $L_{z}=3$ nm is fixed (Figure 8b). The transition energy decreases more rapidly because increasing *L* simultaneously relaxes the confinement along the *x* and *y* directions. As *L* becomes large, the systems approach a quasi-two-dimensional regime in which the confinement is primarily determined by the vertical dimension. The asymptotic transition energies are approximately 2.16 eV for the box and cylinder, 1.94 eV for the ellipsoid, and 1.80 eV for the pyramid. The latter value is particularly close to the CdSe bulk band gap of 1.75 eV, indicating substantially weaker residual confinement. Similarly to Figure 8a, as *L* increases, the energy contribution associated with confinement along the *z*-direction becomes increasingly relevant. Since the box and the cylinder have exactly the same height $L_{z}$, their exciton transition energies are expected to converge, as is clearly observed (see the black and red curves in Figure 8b). An important feature is the reversal of the pyramidal behavior between the two parameter variations. The pyramid has the highest transition energy when $L_{z}$ is varied, but the lowest transition energy when *L* is increased. This results from its geometry, with height $h_{p}=3L_{z}$. In Figure 8a, the fixed L=3 nm maintains strong lateral confinement, whereas in Figure 8b, the fixed $L_{z}=3$ nm gives the pyramid a height of 9 nm, already larger than the corresponding dimensions of the other structures. Consequently, increasing *L* produces a structure with comparatively weak overall confinement. This result highlights that the exciton transition energy is governed not only by the total volume of the QD but also by how that volume is distributed in space. Overall, Figure 8 demonstrates that the geometry and aspect ratio provide effective parameters for tuning the exciton transition energy. Although the four QDs have the same volume and cross-sectional area for each pair of *L* and $L_{z}$, their transition energies differ substantially, particularly in the quasi-one-dimensional and quasi-two-dimensional limits. The results therefore confirm that shape-induced changes in carrier confinement and Coulomb interaction can significantly modify the optical response of nanoscopic CdSe QDs.

Note the inversion behavior for the pyramid by comparing Figure 8a,b; this is due to the fact that when $L_{z}$ is varied at fixed L=3 nm, its height increases as $h_{p}=3\;L_{z}$, while its transverse section continuously decreases toward the apex. This nonuniform cross section prevents the carriers from exploiting the entire longitudinal extension as efficiently as in structures with uniform cross section and keeps the ground-state densities concentrated toward the broader part of the pyramid. Consequently, the pyramid retains comparatively strong effective confinement and exhibits the highest transition energy. In contrast, when *L* is increased at fixed $L_{z}=3$ nm, the pyramidal height remains $h_{p}=9$ nm while its base expands strongly. The lateral confinement then becomes progressively weaker, while its vertical extension remains substantially larger than that of the box and cylinder. As a consequence, the pyramid approaches a weakly confined configuration more rapidly and ultimately exhibits the lowest transition energy. This explains the inversion as a direct consequence of the anisotropic geometric scaling and the associated redistribution of the electron and hole densities.

## 4. Conclusions

In this work, we investigated the electronic and excitonic properties of a single exciton confined in CdSe QDs with box, cylindrical, ellipsoidal, and pyramidal geometries using a self-consistent Schrödinger–Poisson approach within the effective-mass and Hartree approximations. Imposing equal volume and cross-sectional area allowed the effects of geometry and aspect ratio to be isolated from those of the overall QD size.

Increasing either $L_{z}$ or *L* redshifts the excitonic transition, with a stronger variation for *L*. As the structures evolve toward the quasi-1D and quasi-2D regimes, the transition energy becomes governed by the confinement along the directions that remain restricted. This explains why the cylinder and ellipsoid, which share the same circular cross-sectional area, converge to similar transition energies at large $L_{z}$: the transverse confinement in the xy plane dominates, while the electron–hole Coulomb interaction, although it contributes to the absolute energies, is not the origin of this convergence. The Coulomb interaction decreases as the carrier distributions spread and becomes more geometry-dependent away from the strongly confined 0D regime. The pyramid shows the most distinctive behavior: it has the highest transition energy when $L_{z}$ is varied but approaches the lowest when *L* becomes large, an inversion arising from its distinct geometric scaling.

Overall, shape and aspect ratio provide effective means of tuning exciton transition energies. Even for identical volumes and cross-sectional areas, different boundaries impose different confinement conditions and carrier distributions, so the spatial distribution of the volume is as important as the volume itself. The results for large aspect ratios should be interpreted as geometry-dependent trends within the Hartree approximation, since explicit electron–hole correlations, increasingly relevant outside the strong-confinement regime, are not included in the present model.

## Figures and Tables

**Table 1:** List of parameters used.

Parameter	Value
*ℏ*	1.0546 × $10^{-34}$ J s
$m_{0}$	9.1094 × $10^{-31}$ kg
$m_{e}^{*}$	0.13 $m_{0}$ [57]
$m_{h}^{*}$	0.45 $m_{0}$ [57]
$\varepsilon_{0}$	8.8542 × $10^{-12}$ F/m
$\varepsilon_{r}$	9.3 [58]
*e*	1.6022 × $10^{-19}$ C
$E_{g}$	1.75 eV [59]

**Table 2:** Comparison of the calculated and experimental transition energies.

*R* (nm)	$E_{X}^{\exp}$ (eV)	$E_{X}^{\mathrm{theory}}$ (eV)	ΔE (eV)	Error (%)
2.1	2.55	2.54	0.01	0.39
2.6	2.25	2.25	0.00	0.00
3.8	2.06	1.97	0.09	4.37

**Table 3:** Geometrical criteria for crossover between quantum-wire and nanoplatelet regimes.

Regime	Geometrical Criteria	Parameter Range
0D	$L_{z}/L∼1$	$L=L_{z}=3$ nm
quasi-1D	$L_{z}/L≳4$	$L_{z}≳12$ nm, L=3 nm
quasi-2D	$L/L_{z}≳7-8$	L≳ 20–25 nm, $L_{z}=3$ nm

## Data Availability

The data presented in this study are available on request from the corresponding author due to institutional restrictions on public data sharing.
